# Twelve-Month Review of Infusion Pump Near-Miss Medication and Dose Selection Errors and User-Initiated “Good Save” Corrections: Retrospective Study

**DOI:** 10.2196/20364

**Published:** 2020-08-11

**Authors:** James Waterson, Rania Al-Jaber, Tarek Kassab, Abdulrazaq S Al-Jazairi

**Affiliations:** 1 Medication Management Solutions Becton, Dickinson & Company, LLC Dubai United Arab Emirates; 2 Pharmaceutical Care Division King Faisal Specialist Hospital & Research Centre Riyadh Saudi Arabia

**Keywords:** medication library, smart infusion pumps, near-miss error, medication safety, lookalike-soundalike

## Abstract

**Background:**

There is a paucity of quantitative evidence in the current literature on the incidence of wrong medication and wrong dose administration of intravenous medications by clinicians. The difficulties of obtaining reliable data are related to the fact that at this stage of the medication administration chain, detection of errors is extremely difficult. Smart pump medication library logs and their reporting software record medication and dose selections made by users, as well as cancellations of selections and the time between these actions. Analysis of these data adds quantitative data to the detection of these kinds of errors.

**Objective:**

We aimed to establish, in a reproducible and reliable study, baseline data to show how metrics in the set-up and programming phase of intravenous medication administration can be produced from medication library near-miss error reports from infusion pumps.

**Methods:**

We performed a 12-month retrospective review of medication library reports from infusion pumps from across a facility to obtain metrics on the set-up phase of intravenous medication administration. Cancelled infusions and resolutions of all infusion alerts by users were analyzed. Decision times of clinicians were calculated from the time-date stamps of the pumps’ logs.

**Results:**

Incorrect medication selections represented 3.45% (10,017/290,807) of all medication library alerts and 22.40% (10,017/44,721) of all cancelled infusions. Of these cancelled medications, all high-risk medications, oncology medications, and all intravenous medications delivered to pediatric patients and neonates required a two-nurse check according to the local policy. Wrong dose selection was responsible for 2.93% (8533/290,807) of all alarms and 19.08% (8533/44,721) of infusion cancellations. Average error recognition to cancellation and correction times were 27.00 s (SD 22.25) for medication error correction and 26.52 s (SD 24.71) for dose correction. The mean character count of medications corrected from initial lookalike-soundalike selection errors was 13.04, with a heavier distribution toward higher character counts. The position of the word/phrase error was spread among name beginning (6991/10,017, 69.79%), middle (2144/10,017, 21.40%), and end (882/10,017, 8.80%).

**Conclusions:**

The study identified a high number of lookalike-soundalike near miss errors, with cancellation of one medication being rapidly followed by the programming of a second. This phenomenon was largely centered on initial misreadings of the beginning of the medication name, with some incidences of misreading in the middle and end portions of medication nomenclature. The value of an infusion pump showing the entire medication name complete with TALLman lettering on the interface matching that of medication labeling is supported by these findings. The study provides a quantitative appraisal of an area that has been resistant to study and measurement, which is the number of intravenous medication administration errors of wrong medication and wrong dose that occur in clinical settings.

## Introduction

### Background

Infusion programming is a far more complex process than oral medication administration, and it frequently involves the administration of medications from the highest risk groups [1], including heparin, insulin, sedatives, opiates, and critical short half-life medications such as norepinephrine and dopamine [2]. While some work has been done on the role of smart pumps that are capable of reporting their status to centralized monitoring systems to help ensure maintenance of critical short half-life infusion [3] and on the role of medication library hard and soft dose limits during set-up and during titrations of medications [4], it has been generally accepted that even with aggressively managed medication libraries, extensive and ongoing training, and compliance monitoring, only 28% of intravenous (IV) medication errors can be averted with dose error reduction software (DERS) alone [5], as DERS cannot detect errors of right patient, right medication, right order, right documentation, right therapy, and right time [5].

The current paper challenges this assumption to some degree. Our first hypothesis is that many potential lookalike-soundalike (LASA) errors made during medication selection from the pump’s medication library may be prevented by the presence of full names, large characters, and TALLman medication displays on the pump during programming, that wrong dose selection may also be reduced by the presence of standardized concentrations, and that concentration limits built into the pump’s DERS will also catch a high number of “death by decimal point” errors [6].

In one observational study [7], in a high-fidelity simulation laboratory designed to assess the impact of infusion pump technologies (comparing a traditional pump, smart pump, and smart pump with a barcode reader) on nurses’ ability to safely administer intravenous medications, nurses remedied “wrong patient” errors more often when using the barcode pump (88%) than when using the traditional pump (46%) or the smart pump (58%). The barcode pumps were not integrated into the electronic medical record (EMR); therefore, the nurses’ remedial changes were entirely based on a visual check between the pump screen and the patient’s ID wristband of what was either manually entered as patient ID or populated on the pump via scanning of the patient’s ID wristband. Essentially, having to undertake patient identification verification on the barcode pump greatly increased the nurses’ resolution of the “wrong patient” error (the patient identification armband on the mannequin did not correspond to the patient information on the physician order). We suggest that clear and well-presented information on a smart pump screen, which can be verified against other identifiers (in the case of the study facility medication name and dose are clearly printed on each medication in the pharmacy [not handwritten]), may lead to “good catches” of errors during programming of smart pumps for administration of IV medications.

We also recognize that among all of the parts of the medication chain (from prescription to administration), intravenous medication errors, which occur at the point of administration, are the hardest to detect and that in terms of failure mode effect analysis (FMEA), the process consistently scores as a high-risk activity by virtue of the score for “likelihood of detection,” with a high score commonly being applied by organizations utilizing FMEA (scale: 0 [minimum] to 10 [maximum]) [8,9].

Our second hypothesis is that analysis of smart pumps’ DERS logs for near-miss wrong medication or wrong dose selections will help to further extend our understanding of the incidence of these administration errors.

This is important as the existing methods of assaying IV medication administration error and general medication administration failure in any of the general “administration rights” (right patient, right medication, right order, right documentation, right therapy, and right time) are limited and cannot give an accurate idea of the extent of the problem. For example, in one study, an extensive chart review found 398 adverse drug (medication) events (ADEs) at the administration stage, while in the same time period, voluntary reports via the hospital’s anonymous ADE and near-miss event reporting system detected only 23 events [10].

This needs to be viewed against quantitative evidence from what we can see of the iceberg. In a study of voluntary and near-miss reporting of errors in pediatric patients and neonates, which lasted for 1 year, it was found that of 989 reported medical errors, 401 (40.5%) were related to medication. Additionally, 88.0% (353/401) of these errors reached the patient and 33.4% (118/353) of the dose-related errors were related to administration. Moreover, 13.2% (53/401) of errors were of omission [11].

In one well-constructed study of self-reporting by nurses and physicians, the observed rate of parenteral medication administration errors per 100 patient days was 74.5, with 12 patients (0.9% of the total study population) experiencing permanent harm or death [12]. Of course, deriving metrics from self-reporting will always underestimate the frequency and consequences of errors, as many will be undetected by the user. We suggest that adding quantitative data pertaining to medication and dose selection by users, which are derived from smart pump medication library logs, will help shed further light on the murky area of point of care IV medication administration.

Attempting a more accurate “count” of the IV medication administration error rate, owing to its impact on costs, length of stay, and treatment of any sequelae, is, of course, central to delivering value-based health care [13] and to creating a systematic approach for patient safety. It also speaks directly to a central issue in modern health care, that is, cost benefit, as systems employing interoperability between the patient’s EMR and bidirectional interoperable smart pumps for closed-loop bidirectional IV pump-EMR autopopulation utilizing barcode medication administration require extensive investment, but are capable of mitigating wrong time, omitted medication, wrong patient, wrong medication, and wrong dose-type administration errors [14]. Thus, while these systems have been shown to reduce self-reported safety events related to infusion pump programming by a ratio of 3:1 [15] and it has been suggested that “until barcode pumps are integrated with other systems within the medication administration process, their role in enhancing patient safety will be limited” [7], it would be of great value to have a “harder” number for wrong medication and wrong dose-type administration errors from preimplementation data to more concretely prove the economic value of the solution of bidirectional IV pump-EMR autopopulation utilizing barcode medication administration.

Similarly, the documentation available in smart pump event logs and DERS library records has not previously been extensively used as a comparative tool to routinely check the veracity of the medication administration record and is commonly only used in the case of sentinel events. Autodocumentation of continuous infusion and intermittent medications administered via smart pumps directly in the patient’s record is certainly superior to manual completion of the medication record, as manual infusion documentation may be delayed or inaccurate because clinicians attend to emergent situations or have distractions [16]. Once clinicians return to their documentation after a patient care event, such as medication administration, they often transcribe from memory. It would be useful to have the ability to rapidly compare and contrast information derived from the smart pump’s library data to manual chart entries.

### Objectives

The overall objective of this study was to establish, using an easily reproducible and reliable methodology, baseline data to show how metrics in the set-up and initial programming phase of intravenous medication administration can be produced from review of medication library “near-miss” reports from infusion pumps used in varied disciplines and care areas across large facilities with many thousands of IV pumps.

Of particular interest were user-initiated corrections of the more common “death by decimal point” errors of incorrect dose or concentration selection and corrections of wrong medication selection, which is often related to medication name LASA issues. The study also focused on the time taken by clinicians to correct these set-up errors.

Two hypotheses were decided upon at the outset of the study as follows:

1. We hypothesized that potential LASA errors during medication selection in a smart pump’s medication library may be greatly reduced by the presence of full names, large characters, and TALLman medication displays on the pump during programming, that wrong dose selection may also be reduced by the presence of standardized concentrations, and that concentration limits built into the pump DERS will catch a high number of potential “death by decimal point” errors.
2. We hypothesized that analysis of smart pumps’ DERS logs for near-miss wrong medication or wrong dose selections will help to further extend our understanding of the incidence of these administration errors and add quantitative measurement to a process that has, up to now, only been assayed with self-reporting of near-miss errors and recognized errors, simulation laboratory studies, chart reviews, and observational studies, all of which have inherent weaknesses.

## Methods

### Study Design

We undertook a 12-month retrospective review of medication library near-miss error report logs from 2044 wireless-connected modular infusion pumps (846 syringe driver modules, 3662 large-volume pump modules, and 62 patient-controlled analgesia modules [one modular infusion pump can accommodate a mix of up to four syringe, large-volume, or patient-controlled analgesia modules]) used in 15 disciplines/care areas across a large facility with 1852 inpatient beds and 12,601 inpatient admissions yearly, which serves the heart of metropolitan Riyadh, in order to obtain metrics on the set-up phase of intravenous medication administration. The DERS used in this study records any attempt by the user to use a dose outside of the accepted hospital formulary range for each medication. A particular feature of the DERS used in this study is that it records all cancelled infusions, medication concentration limit breaches, and resolutions of infusion alerts by the user. Date-time stamps are automatically applied to all of these alerts and actions by the device.

Data are continually collected from the smart pump logs in our facility, and all nursing and medical staff are aware of this ongoing collection and analysis of near-miss events, as the DERS library itself was created and is updated through a multidisciplinary team feedback mechanism as part of our facility-wide process of Joint Commission International (JCI) quality improvement, Magnet accreditation, and zero-harm targets. The smart pump DERS library data are constantly available to the pharmacy department, and according to the facility protocol, the pharmacy department owns the data and is recognized as the lead department for medication safety. While nursing and medical staff are aware that data are constantly obtained on good catches in medication safety, they were not informed that a particular period would undergo a deeper analysis beyond standard quarterly reviews. This is important as we wanted to get as close as possible to “normal behavior” with our data. As with all observational and self-reporting studies, the Hawthorne effect is a very real danger, and the advantage of “passive” data collection, such as collection in this study, is that users will not alter their behavior as they might during a time-limited study.

There is a regular process of engagement with nursing leadership and clinical educators to provide feedback on good catches, compliance levels, and the need for functional changes to the DERS library as part of the hospital’s zero-harm program and ongoing Magnet and JCI accreditation processes. The risk-management committee for IV medication therapy in the facility will be appraised of the implications of the study with regard to proposed moves to IV medication interoperability and barcode medication administration.

An analysis was undertaken using patient anonymized data for infusions in all areas of the facility. Decision times of clinicians were calculated from the time-date stamps of the pumps’ DERS logs (the pump logs report in hh:mm:ss). The pumps are wirelessly connected to a central server that maintains universal and accurate time keeping for all connected devices. The wireless connectivity also allows for pumps in all areas of the facility to be updated regularly and rapidly with current medication libraries and allows for continual download of medication library and clinician performance, as well as library compliance data.

The study was limited to one pump brand (BD Alaris^TM^ System 8015LS PC Units with Guardrails^TM^ 9.33 DERS software). These smart pumps are connected to a central server (BD Alaris^TM^ Systems Manager) that allows for wireless deployment of medication libraries to the pumps and continuous medication library performance data download from them to a central SQL database, which can be accessed via reporting software (BD Alaris^TM^ CQI Reporter 10.17). These pumps are modular, and each PC unit can carry a mix of up to four large-volume pumps, syringe pumps, or patient-controlled analgesia pumps. All these modular pumps share a common DERS. The DERS has maximum hard limits for dose and duration/rate, above or below which the clinician cannot titrate or set-up an incorrect delivery dose (rate or concentration), and maximum and minimum soft limits, which when breached give an alert to the clinician, who must then decide whether to override the warning. Each distinct group of events from the first alert to resolution is tied together by a unique sequence identification number.

Within the Guardrails^TM^ DERS, the pharmacist may create up to 10,000 medication set-ups with 30 care areas or “profiles” carrying medications and concentrations specific to the care area. Medications may also be set up with free text entry for the clinician at the point of care for dose and volume. These free text dose and volume entries can be limited with concentration limits, which require that any entries are within the minimum and maximum limits for dose/mL. Each profile can also have hard limits placed for maximum patient weight and body surface area.

A DERS master library contains a standard list of medications that can be added with new medications. The DERS master library will accept free text entries for medication names. The maximum character count for each medication entry is 20 characters.

The Guardrails^TM^ software present in these devices allows for the creation of “therapies” that allow the clinician to select the medication name and then select a specific usage for which the dose limits, duration, or rate may differ according to specific indications. For intermittent infusions, specific therapeutic durations and individual weight-based dosing and body surface area–based dosing can be added for each use of a specific medication. Table 1 presents examples of continuous and intermittent infusion therapies.

If the “therapy” option is utilized, each medication may be identified in up to 20 characters, and the therapy listed below the medication name can also be identified by a further 20 characters. In this study, the therapy option was active in all care areas and used extensively in the oncology department’s profile.

Several treatment options for individual medications were also present as separate entities in the libraries, and examples are presented in Table 2.

**Table 1:** Examples of therapies.

Core medication	Therapy title	Variations
Midazolam	Short-term vent	Continuous and bolus dose limits
Midazolam	Conscious sedation	Continuous and bolus dose limits
Midazolam	Status epilepticus	Continuous and bolus dose limits
Cisplatin	Cisplatin 10 mg/m^2^/24 h	Dose by BSA^a^ and by duration (for different oncology regimens)
Cisplatin	Cisplatin 100 mg/m^2^/2 h	Dose by BSA and by duration (for different oncology regimens)
Cisplatin	Cisplatin 25 mg/m^2^/1 h	Dose by BSA and by duration (for different oncology regimens)

^a^BSA: body surface area.

**Table 2:** Examples of individual entities for medications.

Core medication	Treatment option	Variations
Amiodarone	Amiodarone load	Dose and rate/duration
Amiodarone	Amiodarone maintenance	Dose and rate/duration
Alteplase	Alteplase loading	Duration
Alteplase	Alteplase 0.5 mg/mL	Duration
Amphotericin	Ampho B (liposomal)	Dose and rate/duration
Amphotericin	Amphotericin B	Dose and rate/duration
Insulin (Actrapid)	Insulin hyperkalemia	Dose total and rate/duration
Insulin (Actrapid)	Insulin continuous	Dose total and rate/duration
Heparin	Heparin low dose	Maximum dose/hour
Heparin	Heparin high dose	Maximum dose/hour

The DERS can also present clinical advisories after the medication selection has been made, giving specific information about the medication to be administered, such as observations to be made during administration, intravenous administration line type, and specific precautions. Acknowledgement of a clinical advisory must be made by the clinician before the pump allows progress through the programming sequence. Textbox 1 presents examples of clinical advisories.

The pumps are capable of bidirectional communication with the EMR and have the capability to have orders sent directly via wireless technology from the EMR to the pump, thus reducing manual programming and allowing for bidirectional IV pump-EMR autopopulation utilizing barcode medication administration of the pump and autodocumentation of medication delivery. No pumps in this study were connected to the EMR.

**Examples of clinical advisories.**

Clinical advisories requiring confirmation/acknowledgement by the clinician

- 0.22 micron filter required

- Via central line only

- For patient 60 kg or less

- For hyperkalemia

- Loading dose

### Study Procedure

The data were patient anonymized, and no personal information items, such as hospital number, gender, name, date of birth, diagnosis, and other identifiable material, were recorded for analysis.

The BD medical affairs department was engaged for a deeper analysis of the data than is undertaken in our standard quarterly reviews. The BD medical affairs department operates as a distinct arm outside of the commercial operations of the company.

### Inclusion Criteria

All infusions started from within the medication library (and therefore identifiable in terms of medication name selection, medication dose selection, and medication concentration selection) over the 12-month period were included in the study. These included continuous and intermittent infusions, weight-based and nonweight-based infusions, and body surface area–based infusions.

### Exclusion Criteria

Infusions started from outside of the medication library using the “basic infusion” (mL/h) option, which does not record medication name or dose data for the infusion, and medications run through the pumps’ medication calculation option, which also does not record medication name data, were excluded from the study. The DERS and reporting software used in the study allowed for a rapid appraisal of compliance with the medication library in percentage terms from all care areas in the study facility. This metric was included in the study as a check for the veracity of the data included.

## Results

Compliance with medication library usage was 74.29% (1,050,531/1,414,191) of all infusions given in the 12-month period across the facility, and this allowed for a high volume of identifiable infusions to be entered into the study. Intravenous medications (continuous and intermittent) and intravenous fluids (plain and with additives) were present in the library.

Cancelled infusions represented 15.37% (44,721/290,807) of all medication library alerts (Table 3), making them more common than hard-limit alerts that are designed to prevent potentially lethal overdoses.

Within the cancelled infusion group, wrong medication selection represented 22.40% (10,017/44,721) of the cancelled infusions. Among these cancelled medications, all high-risk medications, oncology medications, and all IV medications delivered to pediatric patients and neonates required a two-nurse check according to the local policy. Wrong dose selection was responsible for 19.08% (8533/44,721) of infusion cancellations. A total of 603 infusions were cancelled in response to a concentration limit alert. These are always related to so-called “wildcard” or custom concentrations [6]. In the medication library used in this study, these alerts are captured under the group “reprogram limit alert” (Table 4).

**Table 3:** All medication library alerts by type.

Alert type	Value (N=290,807), n (%)
Reprogram limit alert (hard limit)	40,184 (13.81)
Override limit alert (soft limit)	141,474 (48.65)
Cancelled infusion	44,721 (15.37)
All other alerts	64,428 (22.17)

**Table 4:** Incidences of the causes for cancellation of infusion.

Cause	Percentage of cancelled infusions	Percentage of all medication alerts	Value (N=44,721), n	Comments
Incorrect medication selected	22.40	3.45	10,017	See note on medication name and position of the LASA^a^ error.
Wrong dose selected	19.08	2.93	8533	See note on factor of error.
Indeterminate cause	58.46	8.99	26,144	No evidence of dose error or LASA medication selection error. Possible causes: - IV^b^ access failure - Patient condition change - Therapy discontinuation - Infusion administration backlog with limited IV access
Wrong channel selected	0.04	—^c^	17	Medication for patient-controlled analgesia initially loaded in syringe driver.
Dose cancelled	0.02	—	10	Drug library exited and drug calculator utilized.
Concentration limit breached	N/A^d^	0.21	603	Captured in “reprogram limit alert”

^a^LASA: lookalike-soundalike.

^b^IV: intravenous.

^c^Value is too small to report.

^d^N/A: not applicable.

In terms of the error factor for dose corrections, generally, the potential overdose was not substantial (median 1.5 times the corrected dose); however, the mean (14.52, SD 57.89) was skewed by some very large outliers, as there were 11 corrections made with a dose error factor greater than 100 times the corrected dose (maximum was 500 times the corrected dose).

The average error recognition to cancellation and correction times were 27.00 s (SD 22.25 s, maximum 113 s, minimum 4 s, median 21 s) for medication error correction and 26.52 s (SD 24.71 s, maximum 116 s, minimum 6 s, median 19 s) for dose correction.

It is notable among the results that the difference between the second attempt (and presumably correct) drug selection and the first selection was more prevalent for misidentification in the beginning of the medication’s name, but there was also a substantial number of middle and end name errors being corrected. Examples are provided in Tables 5 and 6.

**Table 5:** Examples of cancelled infusion medication names and corrected medication names.

Cancelled drug/fluid	Final drug/fluid	Key letter position
Sodium bicarbonate	Sodium phosphate	Name end (3)
Abatacept <60 kg	Acetaminophen	Name beginning (1)
Acetylcysteine	Acyclovir	Name middle (2)
Ceftazidime	Ceftriaxone	Name middle (2)
Flucloxacillin	FLUconazole	Name middle (2)
Calcium chloride	Calcium gluconate	Name middle (2)
Cefazolin	Ceftazidime	Name middle (2)
Ceftazidime-Continuo	Ceftazidime-extended	Name end (3)
0.45% NS	0.9% Normal saline	Name beginning (1)
Insulin high non-ICU	Insulin hyperkalemia	Name middle (2)

**Table 6:** Incidence by word/phrase error position.

Word/phrase error position	Incidence (N=10,017), n (%)
Name beginning (1)	6991 (69.79)
Name middle (2)	2144 (21.40)
Name end (3)	882 (8.81)

## Discussion

An extensive study of errors in critical care concluded that “most serious medication errors in critical care occur during the execution of treatment, with performance-level failures outweighing rule-based or knowledge-based mistakes” [17]. This conclusion is supported by our findings. Furthermore, it is evident that smart pump libraries with dose limits can prevent performance-level errors in terms of serious set-up errors that can lead to classic “death by decimal point” errors, such as the 11 near-miss errors of doses greater than 100 times the corrected value. The study also indicates that thorough and scrupulous attention to detail when creating the DERS library for smart pumps can improve patient safety. By example, the number of concentration limit breaches in our study was small and certainly far smaller than that suggested in a 2018 United States survey of the use and application of this DERS safety net, with only 50% of practitioners reporting understanding the value of a hard stop for minimum concentration limits and almost half of all respondents, including 29% with direct responsibility for DERS libraries, being confused by the question or unsure whether their pumps had a hard stop for minimum concentration limits for custom concentrations [6]. This is probably related to the extensive use of standardized concentrations in the facility and the avoidance of wildcard or custom concentrations through alignment from the formulary and computerized prescription order entry system to the smart pump DERS library.

In terms of the average error recognition to cancellation and resolution times being relatively short, with 27 s (SD 22.25) for medication name error correction and resolution and 26.5 s (SD 24.71) for dose correction and resolution, the system in place in the study facility may be an important factor here with all IV medications being prepared and labelled with large clear printing in the central pharmacy, as the medication is “in hand” during programming. This makes it a more effective “independent source of truth” as neither the administering nurse nor the second checker has prepared or labelled the medication to be administered.

What is clearly also important in terms of the recognition and correction of wrong drug name errors at the bedside is that corrections of medication name selection were spread among differences in the beginning, middle, and end of each medication’s name. Older studies on the psychology of reading generally accepted that the beginning and end of words influence readers and tend to make them “guess” the rest of the word, and randomizing letters in the middle of words has little or no effect on the ability of skilled readers to understand text [18]. This is useful for reading at speed, but the deleterious implications of “guessing” for medication safety are obvious as middle letter identification proceeds largely independently of position, and information that the reader gains from the middle letters may operate via the reader using “probability” rather than absolute reading in order to “recognize” the word.

It was chiefly for this reason that the TALLman system of nomenclature was created for LASA medications, and it ensures that “word shape” [19] is disruptive and distinctive for LASA medications. More recent work in cognitive psychology has indicated that when humans read, they use the letters within a word to recognize a word [19]. It was stated that “word shape is no longer a viable model of word recognition. The bulk of scientific evidence says that we recognize a word’s component letters, then use that visual information to recognize a word” [19]. Given what we noted in the spread of the “beginning, middle, and end” of medication names being corrected in this study, it seems reasonable to conclude that more information in terms of letters available to the reader is associated with a higher likelihood of an accurate choice, as the presence of more characters for review is associated with a greater possibility of the reader’s initial instinctive reading (or guessing) being overtaken by “new information” [20]. It is clear that LASA medication errors and near-miss errors are relevant problems for nurses administering IV medications, just as they are for pharmacists dispensing medications, as indicated in a UK survey showing that LASA errors represented 25.9% of total dispensing errors in the last quarter of 2019 [21].

**Figure 1:**
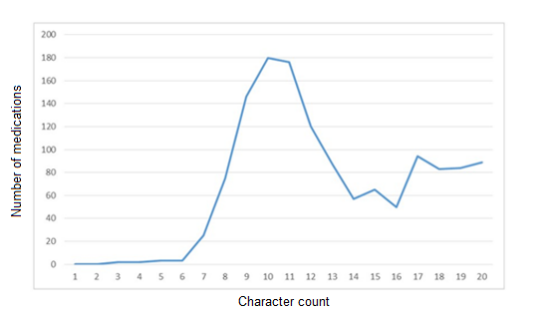
Character count distribution for medication nomenclature in the study facility’s dose error reduction software library.

The intravenous pumps used in this study carry a 20 character maximum, and this maximum capacity was used in many of the medication names in the library. The mean average was 13.04, with a heavier distribution toward higher character counts (Figure 1).

A general recommendation of this study is that intravenous pumps should have character counts of at least 17 characters, given that this was the mean average character count for medications that were corrected by the user. Furthermore, it is recommended that no pump should truncate entire medication entity names during runtime, as this impedes the clarity of information on current infusions required for effective nursing hand-offs.

Given the growth in monoclonal antibody medications in the last few years (518 are currently listed as active medications, with a mean character count of 12 [SD 3.74]) and the fact that we can expect to see an increasing number of these medications, it is worth noting that almost all of these medications end with the suffix “-mab” and have a propensity for using the same or similar name beginnings. Clearly, the need for full naming in medication libraries is critical with these medications. Indeed, in some of these medications, only the second part of their nomenclature differs. Table 7 presents examples of monoclonal antibody naming.

**Table 7:** Examples of monoclonal antibody medications currently in the market, with character counts.

Name	Character count
Cantuzumab mertansine	21
Cantuzumab ravtansine	21
Altumomab pentetate	19
Anatumomab mafenatox	21
Talizumab	9
Tanezumab	9
Trastuzumab	11
Vadastuximab talirine	21
Vandortuzumab vedotin	21

In classic FMEA planning [9], for any high-risk activity, particularly that with a high risk of “low chance or no chance” of error detection, the activity is broken down into a number of steps, each of which can mitigate, correct, or annul any error in the previous steps. The addition of a clinical advisory to known high-risk LASA medications as an extra step in the programming process may therefore be of value. In this study, clinical advisories were commonly used in the oncology profile, as many of these drugs require specific line types. For example, for taxols, the clinician is told via a pop-up advisory screen “paclitaxel: use low-sorbing set with 0.2 micron filter.”

To select this drug, the eight steps for programming (six steps may act to draw the clinician’s attention back to the medication being administered and allow a FMEA stop to be applied) are according to the approach presented in Table 8.

In the adult oncology profile of the medication library, the therapy option, which effectively doubles the character count available to the pharmacist creating the medication library, was used for approximately 60% of all the medications in this profile, with medications, such as carboplatin, having eight distinct therapies and those, such as cisplatin, having thirteen distinct therapies. It was notable that despite the large volume of infusions administered by oncology nurses, the number of wrong medication name errors in the oncology profile was only 55 compared with 322 in the adult general profile and 139 in the adult critical care profile.

**Table 8:** Example of programming a drug associated with a therapy and clinical advisory using failure mode effect analysis steps.

User action	Pump response	FMEA^a^ + action if error detected
CHANNEL SELECT	Presents: Drug library Fluids library Basic infusion	Drug library is the first presented option
GUARDRAILS DRUGS	A-Z in five groups	Can cancel infusion if selection is incorrect
PACLitaxel	Presents therapy options: PACLitaxel 3 weekly PACLitaxel weekly	Can cancel infusion if selection is incorrect
PACLitaxel weekly	PACLitaxel ___mg in ___ml was selected. Is this correct? YES/NO	NO and can cancel infusion if selection incorrect.
YES	Clinical advisory pop up: PACLitaxel: Use low-sorbing set with 0.2 micron filter	NOT CONFIRMED and can cancel infusion if selection is incorrect.
CONFIRM	PACLitaxel weekly User has to complete: ___ mg ___ mL BSA^b^ ___	Can cancel infusion if selection is incorrect.
CONFIRM	PACLitaxel weekly User verifies: Dose Volume BSA Duration (NB^c^ dose/m^2^) is controlled by library limits for this drug, and BSA is controlled by maximum limits per profile. Duration can be default set and controlled according to minimum-maximum in the drug library per drug.	Can cancel infusion if selection is incorrect.
START	Begin infusion	NO START and can cancel infusion if selection is incorrect.

^a^FMEA: failure mode effect analysis.

^b^BSA: body surface area.

^c^NB: nota bene (note well).

General advice from this process would be to ensure that the full name of the medication is given in every step and that it is present in the clinical advisory (this should be a free-text option in smart pumps with this feature).

The JCI organization has noted that half of the cases of preventable harm from medications are associated with the following three categories of medications: opiates, insulin, and heparin [22]. The commission also recommends each facility to create a list from its formulary of LASA medications alongside that of its high-risk medications. A regular review of cancelled infusions and medication name corrections could assist in designing and monitoring the effectiveness of such a strategy. Risk management DERS strategies should aim for a balance of clarity and ease of use, as well as measurement of the usage of the library (compliance). Specialist uses of medications need to be present in therapies, but too many similar options can cause confusion at the bedside. Therapy titling should clearly match the computerized provider order entry system, and this too requires a high capacity character count to be available.

As discussed earlier, the true level of medication administration error for both medication and dose is unknown, despite the best efforts of researchers from every region. It is however clear from the study and from existing literature that the problems of wrong medication selection with LASA medications and wrong dose selection are considerable. It is suggested that bidirectional IV pump-EMR autopopulation utilizing barcode medication administration processes would substantially reduce these two risks to patient safety and also reduce the risk of wrong patient-wrong medication errors. However, bidirectional IV pump-EMR autopopulation is not always deployable for every patient event, as in the case of stat or verbal orders, and there is a need for manual programming in nonnetwork-served areas. Thus, there is still reliance on the local pump-deployed DERS to keep the patient safe, so the principles of full medication name and standardized dose and concentration limits still apply. Furthermore, bidirectional IV pump-EMR integration should only offer autopopulation of smart pumps, as autoprogramming takes too much control away from the clinician at the bedside who may need to hold an infusion for emergent clinical reasons. Indeed, in this study, there were 26,144 cancelled infusions with no specific error identified.

This study makes it clear that how medication information (chiefly name and dose) is presented to smart pump end users using DERS libraries is central to medication safety. An assay of name/dose errors and corrections, particularly for medications used in multiple therapies and with differing dosing, will assist pharmacies in creating safer and more user-friendly DERS libraries. The ability to capture data of near-miss infusion medication errors through wireless systems that can capture every smart pump’s data and to rapidly correct and update DERS libraries across all the facility’s pumps in response to analysis of “what works and what does not” is an important component of any risk management strategy for medication safety, as it quickens the plan, do, check, and act cycle.

## Abbreviations

ADE: adverse drug event
DERS: dose error reduction software
EMR: electronic medical record
FMEA: failure mode effect analysis
IV: intravenous
JCI: Joint Commission International
LASA: lookalike-soundalike
